# Propargyl-Linked Antifolates Are Potent Inhibitors of Drug-Sensitive and Drug-Resistant *Mycobacterium tuberculosis*

**DOI:** 10.1371/journal.pone.0161740

**Published:** 2016-08-31

**Authors:** Behnoush Hajian, Eric Scocchera, Santosh Keshipeddy, Narendran G-Dayanandan, Carolyn Shoen, Jolanta Krucinska, Stephanie Reeve, Michael Cynamon, Amy C. Anderson, Dennis L. Wright

**Affiliations:** 1 Department of Pharmaceutical Sciences, University of Connecticut, Storrs, Connecticut, United States of America; 2 Veterans Affairs Medical Center, Syracuse, New York, United States of America; Purdue University, UNITED STATES

## Abstract

*Mycobacterium tuberculosis* continues to cause widespread, life-threatening disease. In the last decade, this threat has grown dramatically as multi- and extensively-drug resistant (MDR and XDR) bacteria have spread globally and the number of agents that effectively treat these infections is significantly reduced. We have been developing the propargyl-linked antifolates (PLAs) as potent inhibitors of the essential enzyme dihydrofolate reductase (DHFR) from bacteria and recently found that charged PLAs with partial zwitterionic character showed improved mycobacterial cell permeability. Building on a hypothesis that these PLAs may penetrate the outer membrane of *M*. *tuberculosis* and inhibit the essential cytoplasmic DHFR, we screened a group of PLAs for antitubercular activity. In this work, we identified several PLAs as potent inhibitors of the growth of *M*. *tuberculosis* with several of the compounds exhibiting minimum inhibition concentrations equal to or less than 1 μg/mL. Furthermore, two of the compounds were very potent inhibitors of MDR and XDR strains. A high resolution crystal structure of one PLA bound to DHFR from *M*. *tuberculosis* reveals the interactions of the ligands with the target enzyme.

## Introduction

Tuberculosis (TB) is an infectious disease that has affected humans since ancient times. With approximately eight million new cases and one million deaths reported every year, TB remains a major health concern worldwide, ranking among the top few deadly infections [1]. *Mycobacterium tuberculosis* (Mtb), the causative agent of TB in humans, is a slow-growing acid-fast bacterium with a highly impermeable cell wall. Mtb is an opportunistic pathogen that is able to survive within macrophages in a latent form for decades and reactivates in immunocompromised individuals such as those with a concurrent HIV infection [2].

Current treatment for drug-susceptible TB consists of a combination of four medications including isoniazid, rifampicin, ethambutol and pyrazinamide administered for two months followed by four months of treatment with isoniazid and rifampicin [3]. Incompatibility of this regimen with HIV and diabetes medications along with the emergence of multidrug resistant (MDR) and extensively drug resistant (XDR) strains makes treatment even more challenging. MDR-TB strains are resistant to isoniazid and rifampicin, the most effective first-line drugs. Current therapy for MDR-TB consists of a combination of eight to ten drugs administered for one to two years. XDR-TB strains, in addition to isoniazid and rifampicin, are also resistant to fluoroquinolones and at least one of the second-line injectable drugs including amikacin, kanamycin and capreomycin. Treatment of MDR- and XDR-TB is lengthy, expensive, and complex with serious side effects. Therefore, there is an urgent need to develop novel drug regimens that can target MDR and XDR strains, shorten treatment duration, be co-administered with antiretrovirals, and ideally be less toxic and orally available[1, 4–8].

Despite this necessity, the progress of the current clinical pipeline is slow. Bedaquiline, a novel ATP synthase inhibitor [9], is the first new FDA-approved TB drug in 40 years. Some other novel compounds in clinical trials include an oxazolidinone (AZD-5847)[10] that targets the ribosome, SQ-109 a 1,2 diamine, targeting a membrane transporter [11] and bicyclic nitroimidazole PA-824 [12] and benzothiazinone BTZ-043 [13], for which the mechanism of action is not completely known.

Antifolates, compounds that target the folate biosynthetic pathway, have been widely used in medicine as anticancer agents [14], antimicrobials [15], and immunosuppressants [16] and have the potential to become successful antitubercular drugs. The folate pathway plays an essential role in cell survival by generating 5,10-methylene tetrahydrofolate as a one-carbon donor for the synthesis of deoxythymidine monophosphate (dTMP), purines, methionine and histidine. Disruption of this pathway leads to the critical deficiency of these key molecules, impaired DNA replication and ultimately cell death. Dihydrofolate reductase (DHFR) is a critical enzyme in the folate pathway; it is responsible for the NADPH-dependent reduction of dihydrofolate (DHF) to tetrahydrofolate (THF). Although DHFR is a validated drug target for bacterial and protozal infections, it is not currently invoked for TB therapy. Methotrexate, pyrimethamine, and trimetrexate, clinically approved antifolates, are potent inhibitors of the MtbDHFR enzyme but they fail to inhibit the growth of Mtb [17, 18], most likely due to an inability to permeate the lipid-rich cell wall. Designing antifolate compounds that inhibit MtbDHFR enzyme activity and also the growth of live Mtb is a promising strategy for TB drug discovery and development.

Here, we report the activity of a series of propargyl-linked antifolates (PLAs) against the MtbDHFR enzyme and the growth of the live bacterium. We have developed these compounds to inhibit the DHFR activity and growth of various microorganisms such as methicillin-resistant *Staphylococcus aureus* (MRSA) [19–21], *Klebsiella pneumoniae* [22, 23], *Candida albicans* [24–26] and *Streptococcus pyogenes* [27]. Excitingly, several of the compounds potently inhibit the growth of Mtb with MIC values less than 1 μg/mL. We have also evaluated the activity of some of the compounds against the growth of MDR- and XDR-TB strains and show that two of the compounds are very potent inhibitors of the growth of these cells and not subject to cross-resistance with other known mechanisms. Finally, we present a crystal structure of MtbDHFR bound to its cofactor, NADPH and one of the PLAs. Preliminary data reported here suggest that the propargyl-linked antifolates may be good candidates for the design of novel anti-tubercular agents.

## Results

**Fig 1:**
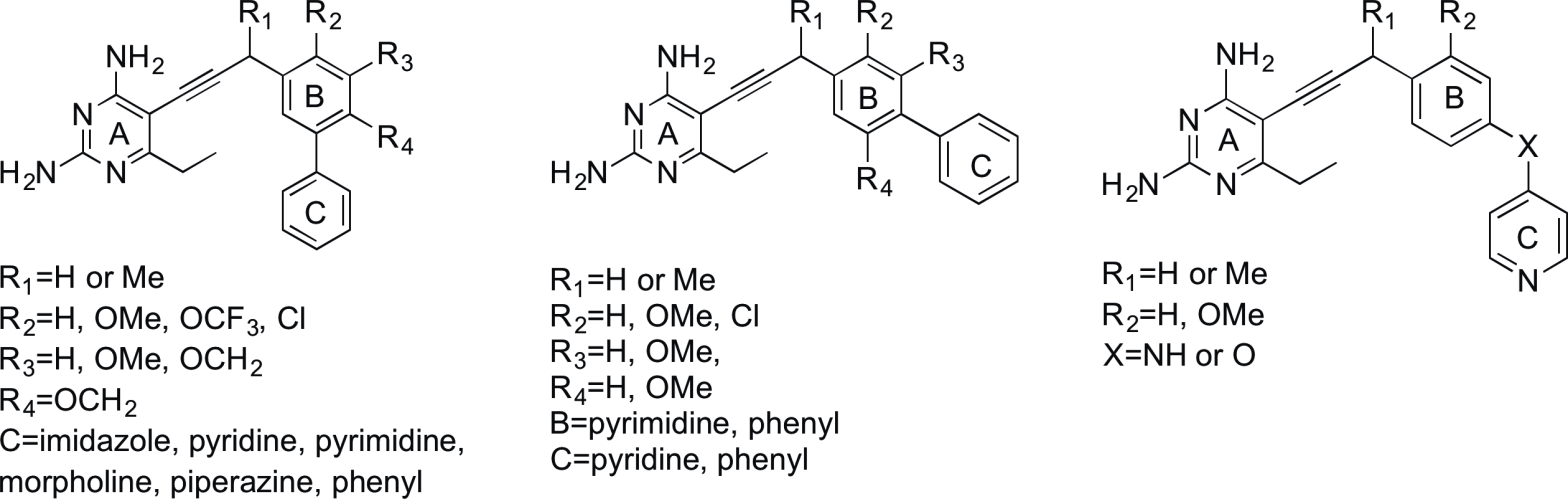
Examples of propargyl-linked antifolates evaluated for antibacterial activity.

**Fig 2:**
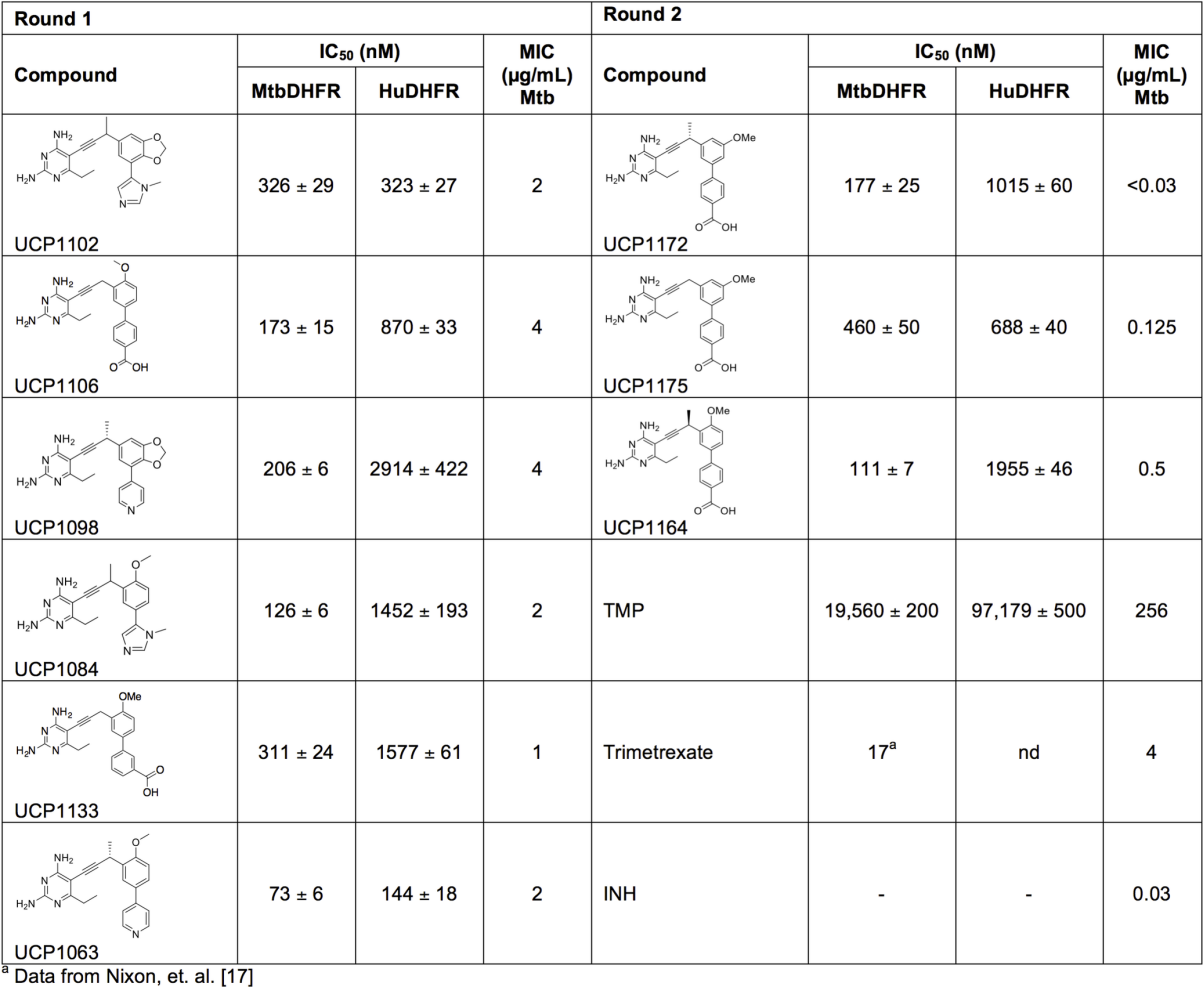
Evaluation of enzyme inhibition and antibacterial activity of propargyl-linked antifolates.

The propargyl-linked antifolates are potent inhibitors of several bacterial DHFR enzymes as well as inhibitors of the growth of several Gram-positive [19–21, 27, 28] and Gram-negative organisms [22, 23]. As we believe the PLAs do not require active transport and enter cells by passive diffusion, it is compelling that they show a capacity to penetrate both of these types of cell walls. With these permeability characteristics in mind, we were motivated to determine if the PLAs inhibit the growth of another bacterium with a complex outer membrane, *M*. *tuberculosis*. We performed a first round of screening with 22 PLAs against the Erdmann strain of Mtb (ATCC 35801). The screened compounds fall into three general subsets: meta-linked, para-linked and bridged-para-linked (generic structures shown in Fig 1). Biological activity and chemical structures for all screened PLAs are shown in Supporting Information (S1 Fig and S1 Table); potent compounds and data are shown in Fig 2. Compounds from all three subseries show promising MIC values (1–4 μg/mL). The first series are meta-linked charged compounds (UCP1106 or UCP1133), benzodioxalane or benzodioxane compounds (UCP1098, UCP1102 and UCP1113) and a group of compounds with a meta-linkage joining rings B and C (UCP1084, UCP1063 and UCP1104). Building on these relationships, a second round of screening explored additional charged[29] compounds. Excitingly, compounds UCP1172, UCP1175 and UCP1164 in this round exhibit very potent MIC values (<0.03, 0.125 and 0.5 μg/mL, respectively) against the Erdmann strain. Variation in the propargylic substitution including hydrogen, methyl or individual enantiomers of a methyl substitution, along with a 2’ or a 3’ methoxy substitution contribute to the differences in MIC values between these compounds.

To validate DHFR as the target that results in cell growth inhibition, eleven compounds were also tested for antibacterial activity in two *M*. *smegmatis* strains that overexpress DHFR under the control of two different promoters (gift from Dr. Martin Pavelka). All compounds, with the exception of UCP1066, have significantly increased MIC values of at least 32 μg/mL in both strains (S2 Table), showing that the antibacterial activity is most likely owing to DHFR inhibition. Compound UCP1066 has an MIC value of 4 μg/mL in the overexpressing strains, which is the same as its value against the Erdmann strain.

We then expressed and purified the *M*. *tuberculosis* DHFR (MtDHFR) protein and measured enzyme inhibition (Fig 2) using standard procedures that spectroscopically follow the oxidation of the cofactor NADPH at 340 nm. Experiments were performed in triplicate and verified on two different dates. Many of the inhibitors tested in our assay are moderately potent against the MtDHFR enzyme with IC_50_ values in the range 70–600 nM. Interestingly, the range of IC_50_ values is relatively narrower than the range of MIC values, again suggesting that activity against the enzyme is only one of the factors that drives antibacterial potency.

We also measured the inhibition of the human DHFR enzyme by each of these compounds. Fig 2 shows that most of the compounds have very similar IC_50_ values against the human enzyme. This concordance in enzyme inhibition results from the overall similarity between the structures of the enzymes and has been noted in other studies examining DHFR inhibition in *M*. *tuberculosis* [18, 30, 31]. In order to investigate potential human cell toxicity, we have examined whether these potent compounds have cytotoxic effects in human dermal fibroblasts, HepG2 and MCF-10 cells. In all cases, the growth of the cells is not inhibited with compound concentrations less than 500 μM[29].

While direct comparison between molecules possessing common features is difficult with this group of inhibitors, there are two highlighted comparisons that show the value of the C-ring COOH substitution. UCP1018, with a 3’-methoxy on the B-ring and a benzodioxane at the C-ring (shown in S1 Fig), has an MIC value of 16 μg/mL whereas the very similar compound UCP1175 with a 3’-methoxy B-ring and phenyl-carboxylate C-ring has an MIC value of 0.125. In another example, UCP1071 has a propargyl methyl, 3’-methoxy B-ring and pyridyl C-ring with an MIC value of 4. Compound UCP1172 with an *R*-methyl at the propargyl position, 3-methoxy and phenyl-carboxylate has an MIC value of <0.03 μg/mL.

As drug resistance is increasing in strains of *M*. *tuberculosis*, the number of efficacious treatment options is decreasing. It is critical that new drug candidates retain significant activity against drug-resistant strains. We further evaluated the most promising lead PLAs (Table 1) against five MDR strains: Mtb 365, Mtb 276, MTb 352, Mtb 56 and Mtb C-31 and one extensively drug-resistant (XDR) strain, Mtb 5. These strains are characterized by a resistance profile shown in S3 Table. Overall, each of the strains are resistant to INH with MIC values between 0.25–4 μg/mL. Three of the strains, Mtb 5, Mtb 365 and Mtb 56, are also highly resistant to rifampin with MIC values of 8, 64 and 32 μg/mL, respectively. The strains are also resistant to a variety of other agents including ethambutol, streptomycin and moxifloxacin (S3 Table). The PLAs are active against the majority of the MDR strains, albeit at slightly decreased levels. The compounds did not show activity against strain C-31, which will be explored in future work to determine if there are mutations in the gene encoding DHFR. Excitingly, compounds 1172 and 1175 are very active against the MDR strain (Mtb 352) and XDR strains (Mtb 5) with MIC values of 0.06 or 0.5 μg/mL, respectively, against Mtb 352 and 0.25 and 2 μg/mL against Mtb 5.

**Table 1:** Antibacterial activity of propargyl-linked antifolates against multidrug-resistant isolates of *M*. *tuberculosis*.

COMPOUNDS	MIC (μg/mL)						
	Mtb Erdman	Mtb 5	Mtb 365	Mtb 276	Mtb 352	Mtb 56	Mtb C-31
**1113**	8	16	8	16	8	8	16
**1106**	16	8	4	16	16	16	>32
**1102**	4	8	8	4	4	8	8
**1098**	8	8	4	8	8	8	16
**1084**	4	4	4	4	4	4	8
**1071**	8	16	8	16	8	8	32
**1066**	8	16	8	16	16	8	32
**1172**	<0.03	0.25	<0.03	ND	0.06	0.06	8
**1175**	0.5	2	0.125	ND	0.5	0.5	8
**INH**	0.125	4	2	ND	1	1	4

It is clear from the results reported here that the ability of a compound to inhibit the enzyme plays only a partial role in the total antibacterial activity. In order to begin to predict the proper physicochemical properties that yield penetration of a compound through the *M*. *tuberculosis* cell wall, we analyzed several physicochemical properties of the PLAs including molecular weight, pKa of key groups, overall charge, logP, logD and polar surface area (PSA; Table 2). The compounds are listed by order of activity from potent to moderately active to inactive. Overall, it appears that active compounds are polar compounds, possessing logD values below 2 and PSA values above 120. There are a few exceptions to this generality, however. Compound UCP1124 has an ortho-carboxylic acid and poor enzyme inhibition, which may account for its poor antibacterial activity. Compound UCP1163 is the opposite enantiomer of UCP1164, which is a potent compound. As both inhibit the enzyme with IC_50_ values of approximately 100 nM, it is not obvious at this point why UCP1163 does not have improved antibacterial activity. Interestingly, a recently published screen of 2508 compounds from the Glaxo antifolate library identified DQn-1, a diaminoquinazoline with an appended carboxylic acid, as the compound with greatest activity (MIC 0.03 μg/mL)[18]. DQn-1 has the following physicochemical properties: molecular weight 343.77, logD 0.81 and PSA 129.98, which are similar to the most potent compounds evaluated in this work. DQn-1 also shares an isoelectric point of 5.5 with our PLA-COOHs which at physiological pH will give an equilibrium where roughly 30% of our PLA-COOHs are charge-neutral zwitterionic. We believe the activity of our PLA-COOHs is largely due to their ability to adopt a zwitterionic form in solution which aids in mycobacterial cell permeation.

**Table 2:** Physicochemical properties of PLAs.

COMPOUND	MW	pKa	CHARGE^a^	logP	logD^a^	PSA	LIPINSKI`S VIOLATION^[^^32^^]^
**UCP1175**	402.45	4.06 (-COOH)	-0.66	4.21	1.66	128.43	0
**UCP1172**	416.48	4.06 (-COOH)	-0.66	4.5	1.95	128.43	0
**UCP1164**	416.48	4.08 (-COOH)	-0.66	4.5	1.95	128.43	0
**UCP1102**	390.18	6.15 (-N)	0.40	2.65	2.45	115.35	0
**UCP1133**	416.48	4 (-COOH)	-0.66	4.50	1.94	128.43	0
**UCP1104**	389.45	11.35 (-NH)	0.34	2.75	2.57	117.40	0
**UCP1098**	387.44	4.55 (-N)	0.34	3.40	3.22	110.42	0
**UCP1084**	376.46	6.42 (-N)	0.44	2.86	2.64	106.12	0
**UCP1106**	402.45	4.08 (-COOH)	-0.66	4.5	1.95	128.43	0
**UCP1142**	377.88	5.01 (-N)	0.35	4.38	4.20	91.96	0
**UCP1071**	373.46	4.32 (-N)	0.34	4.01	3.83	101.19	0
**UCP1066**	374.45	0.72 (-N)	0.34	2.79	2.61	114.08	0
**UCP1063**	373.45	5.16 (-N)	0.35	3.62	3.44	101.19	0
**UCP1171**	421.89	0.68 (-N)	0.34	4.23	4.05	110.42	0
**UCP1165**	431.54	3.63 (-NH2)	0.34	3.79	3.61	123.55	1
**UCP1163**	416.48	4.08 (-COOH)	-0.66	4.50	1.95	128.43	0
**UCP1139**	387.21	9.27 (-NH2)	1.33	3.68	1.65	115.94	1
**UCP1135**	345.41	5.92 (-N)	0.37	3.35	3.15	101.19	0
**UCP1128**	455.48	6.46 (-N)	0.44	5.47	5.23	101.19	1
**UCP1124**	402.45	3.69 (-COOH)	-0.66	4.21	1.64	128.43	0
**UCP1122**	329.4	5.07 (-N)	0.35	3.49	3.31	91.96	0
**UCP1116**	405.92	6.41 (-N)	0.43	4.65	4.41	91.96	0
**UCP1113**	401.46	4.57 (-N)	0.34	3.29	3.11	110.42	0
**UCP1109**	403.48	4.28 (-N)	0.34	3.46	3.28	110.42	0
**UCP1101**	347.18	6.24 (-N)	0.41	1.80	1.60	109.78	0
**UCP1099**	387.44	4.55 (-N)	0.34	3.40	3.22	110.42	0
**UCP1088**	379.49	0.12 (-N), 8.91 (-N)	1.31	2.76	1.08	108.15	0
**UCP1018**	416.18	-	0.34	4.07	3.88	106.76	0
**UCP1170**	476.20	-1.03 (-O), 15.11 (-NH)	0.34	5.73	5.55	108.17	1
**UCP1055**	344.41	1.45 (-N)	0.34	3.74	3.56	104.85	0
**UCP1039**	389.45	4.58 (-N)	0.34	3.18	3.00	110.42	0

^a^ logD and charge were calculated at physiological pH 7.4

**Fig 3:**
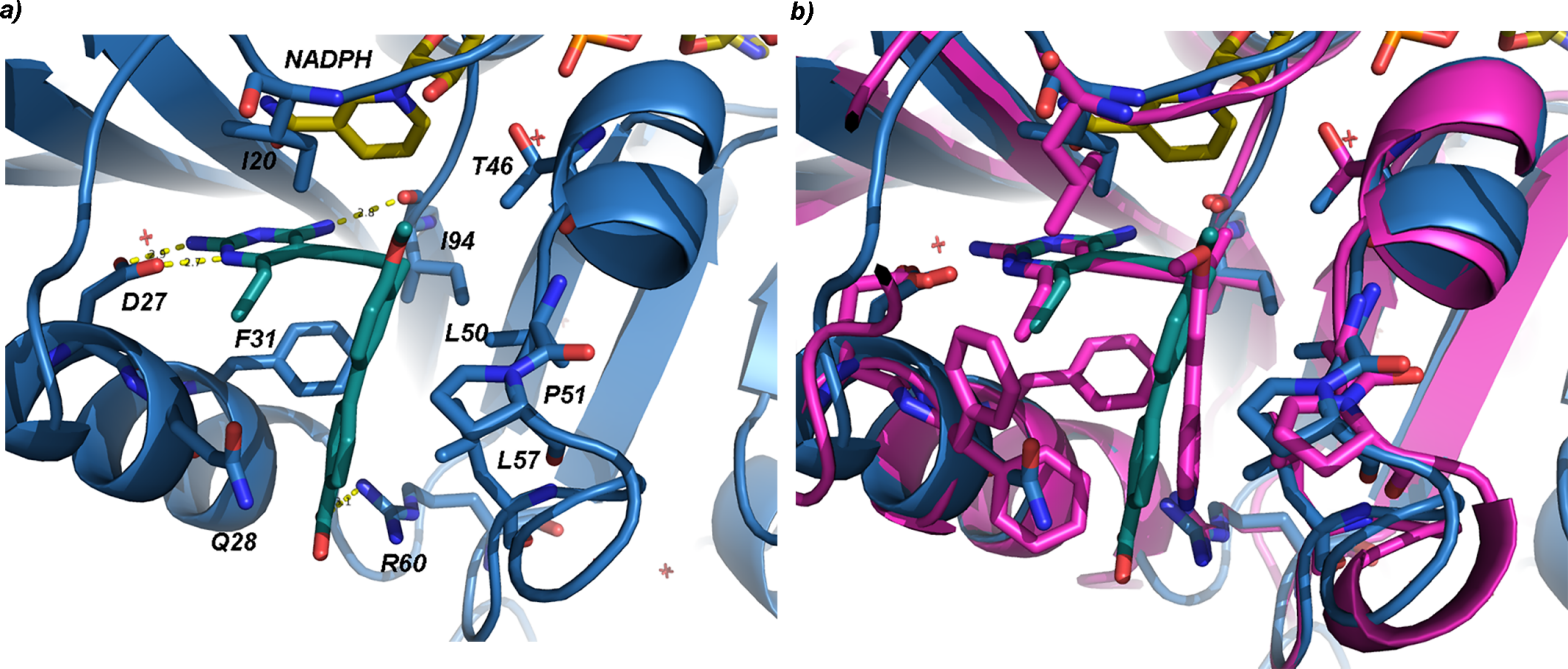
a) Crystal structure of MtbDHFR (blue) bound to NADPH (yellow) and compound UCP1106 (green). Active site residues are labeled. b) Superposition of MtbDHFR: NADPH: UCP1106 (same colors as a) with a structure of human DHFR bound to NADPH and UCP1015 (magenta)[32]. Residue Phe 31 (human structure) has two conformations.

In order to investigate the molecular interactions between MtbDHFR and the PLAs, MtbDHFR was crystallized bound to NADPH and an earlier PLA-COOH compound, UCP1106[29]. The crystals diffracted to 1.8 Å resolution and belong to the P1 space group (Table 3). The complex crystallized with four molecules in the asymmetric unit, providing several independent assessments of the PLA interactions with the enzyme. It is clear that there are several strong ionic and hydrogen bonds between the inhibitor and enzyme that provide a great deal of affinity for the inhibitor (Fig 3A). For example, the diaminopyrimidine ring hydrogen bonds with Asp 27 and a hydrogen bond with Ile 94 in the active site. The carboxylate of UCP1106 forms an ionic interaction with Arg 60. Several van der Waals interactions are also important for binding: Ile 20, Phe 31 and Ile 94 form van der Waals interactions with the diaminopyrimidine ring and the propargyl linker; Leu 50, Pro 51 and Leu 57 form van der Waals interactions with the B and C phenyl rings. The 2’-methoxy on the B-phenyl ring of UCP1106 does not appear to have optimized interactions with neighboring residues, yet the 3’-methoxy on UCP1172 and UCP1175 would be positioned to interact much more favorably with Pro 51.

**Table 3:** Data collection and Refinement Statistics.

	MtbDHFR:NADPH:UCP1106
PDB ID	5JA3
Space group	*P 1*
No. monomers in asymmetric unit	4
Unit cell (*a*, *b*, *c* in Å)	59.612, 60.444, 60.472
Resolution (Å)	34.725–1.814
Completeness% (last shell, %)	72.89 (75)
Unique reflections	55,796
Redundancy (last shell)	1.5 (1.4)
Rsym, (last shell)	0.034 (0.201)
*<I/σ>* (last shell)	28.82 (4.17)
R-factor/Rfree	0.2266/0.2617
No. of atoms (protein, ligands, solvent)	5331
Rms deviation bond lengths (Å), angles (deg)	0.009, 1.366
Average B factor for protein (Å^2^)	33.34
Average B factor for ligand (Å^2^)	32.48
Average B factor for solvent molecules (Å^2^)	30.54
Average B factor for cofactor (Å^2^)	32.57
Residues in most favored regions (%)^a^	95.55
Residues in additional allowed regions (%)^a^	3.97
Residues in disallowed regions (%)^a^	0.48
Collection Location	Stanford Synchrotron Radiation Lightsource

^a^Statistics according to an analysis of the Ramachandran plot

A superposition of a structure of human DHFR bound to NADPH and a PLA [33] with the MtbDHFR complex described here (Fig 3B) shows the striking similarity between the active site residues. Although there is a notable difference with Gln 28 (Mt):Phe 31 (h), the active site residues are largely conserved in identity, conformation and position. The design of inhibitors with polar groups that take advantage of contacts with Gln 28 would potentially yield greater selectivity at the enzyme level. Despite the similarity, some of the lead compounds including UCP1106, UCP1164 and UCP1172 show selectivity toward MtbDHFR (5.0, 17.6, and 5.7 fold, respectively), suggesting that the small differences that do exist can be further exploited for selectivity design.

## Discussion

In this work, several propargyl-linked antifolates were screened for inhibition of the growth of *M*. *tuberculosis*. The PLAs show a range of activity, with several more potent compounds exhibiting MIC values between 1–4 μg/mL. As the PLA-COOH compounds showed the greatest promise overall, a second round of screening revealed three compounds with highly potent activity (MIC values 0.03–0.5 μg/mL). A screen of the most promising lead compounds against MDR and XDR strains revealed that the PLA-COOH compounds maintain their high level of activity, suggesting that they are excellent leads for new drug development efforts against TB. A crystal structure of one of these compounds with MtbDHFR reveals the molecular interactions between the inhibitor and active site.

In general, previous efforts to identify antifolates active against Mtb led to a series of potent enzyme inhibitors that lack significant antibacterial activity. Using a high-throughput screen of 32,000 compounds, an antifolate with a quinazoline ring was identified as an MtbDHFR inhibitor with moderate potency (MIC_99_ = 207 μM) against the H37Rv strain [34]. Another study [31] reports the design and synthesis of a series of 16 diaminotriazines; one of these exhibits an MIC value of 0.325 μM against H37Rv. A third study [30] used a virtual screen to select eight compounds for biological evaluation. All eight show enzyme inhibition with IC_50_ values under 3 μM and one has antibacterial activity between 10–50 μg/mL. In contrast to this difficulty in achieving antibacterial activity, a recent report describing a screen of the Glaxo antifolate collection yielded a potent compound DQn-1 (MIC 0.03 μM) [18], which was also found to inhibit the enzyme and exert on-target antibacterial activity. Importantly, the PLAs described here greatly expand the field of effective antifolates that display significant activity against *M*. *tuberculosis*.

All of the efforts to discover antifolates effective against Mtb build on the fact that antifolates are generally excellent antimicrobial agents but that the ability to permeate the Mtb cell wall is compromised, leading to a lack of antibacterial activity. We and others [35, 36] have attempted to investigate whether key physicochemical properties influence the cell membrane permeability of small molecules. We observed that low values of logD (under 2) and high values of polar surface area (>120) are associated with many of the most potent compounds. Lee also found that logD values between 2.4 and 2.9 and larger values of PSA were desirable for permeability of the Mtb cell wall. In mammalian cells, the increased polarity is expected to decrease permeability. However, the mechanism through which drugs penetrate the mycobacterium cell wall is still poorly developed so it is not clear if the same guidelines are applicable. Recently, an effort to find correlations between a database of antimycobacterial compounds and physicochemical properties led to an available tool for permeability prediction, MycPermCheck [35]. Clearly, understanding the properties of permeable compounds will allow modification of existing inhibitors that are impermeable as well as the selection of new phenotypes that are poised for possessing antibacterial activity.

An important feature of the PLAs is their potent activity against multiply drug-resistant strains. As drug resistance is increasing, it has become critical that new agents are effective at killing existing drug-resistant strains. The compelling activity of compounds UCP1172 and UCP1175 places the PLAs within a small group of compounds that are effective against the wild-type strain, MDR mutants and XDR mutants. These PLA-COOH compounds are clearly excellent candidates for future drug development as antitubercular agents.

## Materials and Methods

### Antimicrobial agents

The synthesis and characterization of compounds UCP1102 and UCP 1084 [23], UCP1098 and UCP1063 [21], and UCP1106, UCP1133, UCP1172, UCP1175 and UCP1164[29] have been described in several publications.

### Drugs

Isoniazid (INH) was purchased from Sigma Chemical Co., St. Louis, MO. and dissolved in 100% dimethyl sulfoxide (DMSO) to a concentration of 1 mg/ml prior to freezing at -20°C. The DHFR inhibitors were dissolved in 100% DMSO to a concentration of 20 mg/ml.

### Isolates

*M*. *tuberculosis* ATCC 35801 (strain Erdman) was obtained from the American Type Culture Collection, Manasas, VA. Clinical isolates were obtained from SUNY Upstate Medical University, Syracuse, NY (provided by Betz Forbes), University of Stellenbosch, South Africa (provided by Tommy Victor), National Center of Tuberculosis and Lung Diseases of Georgia, Tbilisi, Georgia (provided by Natalia Shubladze), National Jewish Center, Denver, CO (provided by Leonid Heifets).

The mycobacterial isolates were grown in modified Middlebrook 7H10 broth (pH6.6; 7H10 agar formulation with agar and malachite green omitted) supplemented with 10% Middlebrook oleic acid-albumin-dextrose-catalase enrichment (Difco Laboratories, Detroit, MI) and 0.05% Tween 80 on a rotary shaker at 37°C for 5–10 days. The organisms were diluted in 7H10 broth to 1 Klett unit (equivalent to about 5 X 10^5^ CFU/ml) using a Photoelectric Colorimeter (Manostat Corp., New York, NY) for use in the broth dilution assay.

### Microtiter broth dilution MIC testing

Polystyrene 96-well round-bottom microtiter plates (Corning Inc., Corning, NY) were filled with 50 μl of modified 7H10 broth. The compounds were prepared at 4 times the maximum concentration at which they were to be tested and then were added to the first well prior to being serially diluted 2-fold. INH was tested using a range of concentrations from 8 μg/ml- 0.008 μg/ml. The DHFR inhibitors were tested using a range of 32 μg/ml to 0.03 μg/ml. The inocula used for each strain were measured by titration and plated on 7H10 agar plates to determine the actual inocula. The 7H10 agar plates were incubated at 37°C for 4 weeks. Fifty microliters of the inocula was added to each well containing compound to yield an initial concentration of about 2.5 x 10^5^ CFU/ml (range for various isolates tested was 1.25 X 10^6^ CFU/ml– 8 X 10^4^ CFU/ml). The microtiter plates were covered with SealPlate adhesive sealing film (Exel Scientific, Wrightwood, CA) and were incubated at 37°C in ambient air for 14–21 days prior to reading. Each isolate was tested in duplicate. The MIC was defined as the lowest concentration of antimicrobial agent yielding no visible turbidity.

### Expression and purification of *M*. *tuberculosis* and human DHFR

BL21(DE3) competent *E*. *coli* cells (New England BioLabs) were transformed with recombinant pET-41a(+) plasmid harboring the *dfrA* gene constructed by GenScript. Transformed cells were grown in LB medium supplemented with 30 μg/mL kanamycin at 37°C until the OD_600_ reached 0.6–0.7. The cells were induced with 1mM IPTG for 20 h at 20°C and spun down at 8000 rpm for 15 minutes. Each gram of wet cell pellet was resuspended in 5 ml of 1X BugBuster reagent (Novagen) supplemented with 200 μg/mL lysozyme and 1mM DNase I (Thermo Scientific). The cell suspension was incubated for 30 minutes at room temperature with gentle rotation followed by centrifugation at 18,000 rpm for 30 minutes and supernatant was collected. In order to precipitate some of non-target proteins, 40% ammonium sulfate was added to the cell lysate and stirred at 4°C overnight. After centrifugation at 18,000 rpm for 15 minutes, the supernatant was passed through 0.22 μm filter and slowly loaded onto a methotrexate-agarose column pre-equilibrated with 4 CV of equilibration buffer A (20 mM Tris-HCl, 50mM KCl, 2mM DTT, 0.1 mM EDTA and 15% (v/v) glycerol, pH 7.5). The column was washed with 3 CV of wash buffer B (20 mM Tris-HCl, 500 mM KCl, 2 mM DTT, 0.1 M EDTA and 15% (v/v) glycerol, pH 7.5). The enzyme was eluted with 3 CV of elution buffer C (20 mM Tris-HCl, 50 mM KCl, 2 mM DTT, 2mM DHF, 0.1 mM EDTA and 15% (v/v) glycerol, pH 8.5). Fractions containing DHFR enzyme, were collected, concentrated and loaded onto a Hi-Prep 26/60 Sephacryl s-200 HR column pre-equilibrated with 1 CV of equilibration buffer A (pH 8.5). The protein elution was monitored with AKTA UV/vis diode array spectrophotometer at 280 nm. Fractions containing pure enzyme were pooled, concentrated to 10 mg/ml and flash frozen in liquid nitrogen and stored at -80°C.

### Enzyme inhibition

Enzyme activity and inhibition assays were performed by monitoring the NADPH-dependent reduction of dihydrofolate catalyzed by the DHFR enzyme. The rate of NADPH oxidation was measured spectrophotometrically at 340 nm in assay buffer containing 20 mM TES pH 7.0, 50 mM KCl, 10 mM 2-mercaptoethanol, 0.5 mM EDTA and 1 mg/mL bovine serum albumin. All measurements were performed at room temperature by adding pure enzyme (2mg/mL), 100 μM NADPH and 100 μM DHF to the buffer. For inhibition assays, inhibitors, dissolved in 100% DMSO, were added to the mixture and incubated for 5 minutes before the addition of DHF. Average IC_50_ values and standard deviations were measured in triplicate.

### Crystallization of *M*. *tuberculosis* DHFR

Crystallization trials were performed using slight modifications of conditions previously reported [37]. Briefly, freshly purified protein in buffer A containing 5% glycerol instead of 15%, was incubated with 5 mM NADPH and 2 mM inhibitor for two hours on ice. After incubation, the protein solution was concentrated to 8 mg/mL and crystallized by hanging drop vapor diffusion method and using EasyXtal 15-well plates (Qiagen). An equal volume of protein:ligand:cofactor complex was mixed with crystallization solution (2 μl +2 μl) and let to equilibrate against 500 μl of well solution at 4°C. Pyrimidal crystals suitable for data collection grew to a size of 0.5 mm x 0.4 mm x 0.3 mm within 8 over 8 weeks from solutions comprising 2.1–2.3 M (NH_4_)_2_SO_4_ and 0.1 M NaAc pH 4.5.

Crystals were flash-frozen in the mother liquor supplemented with 20% glycerol. X-ray data were collected at Stanford Synchrotron Radiation Lightsource (SSRL). Data were indexed and scaled using HKL2000. Structure were solved by molecular replacement using the Phaser [38] using previously reported complex of MtbDHFR:NADPH:trimetrexate (PDB ID: 4M2X). The programs Coot [39] and Phenix [40] were used for structure refinement. The ligand structure and restraints were generated using PRoDRG server [41].

## Supporting Information

S1 Fig Compounds screened against *M*. *tuberculosis*. (TIF)

S1 Table Propargyl-linked antifolates inhibit the Mtb DHFR enzyme and growth of Mtb. (DOCX)

S2 Table MIC values against *M*. *smegmatis* and overexpressing strains. (DOCX)

S3 Table Resistance pattern of organisms tested against DHFR inhibitors. (DOCX)
