# Supplementary figures and images for: Propargyl-Linked Antifolates Are Potent Inhibitors of Drug-Sensitive and Drug-Resistant Mycobacterium tuberculosis
Source: PLoS One. 2016 Aug 31;11(8):e0161740. doi: 10.1371/journal.pone.0161740 (PMC5006990; doi:10.1371/journal.pone.0161740)

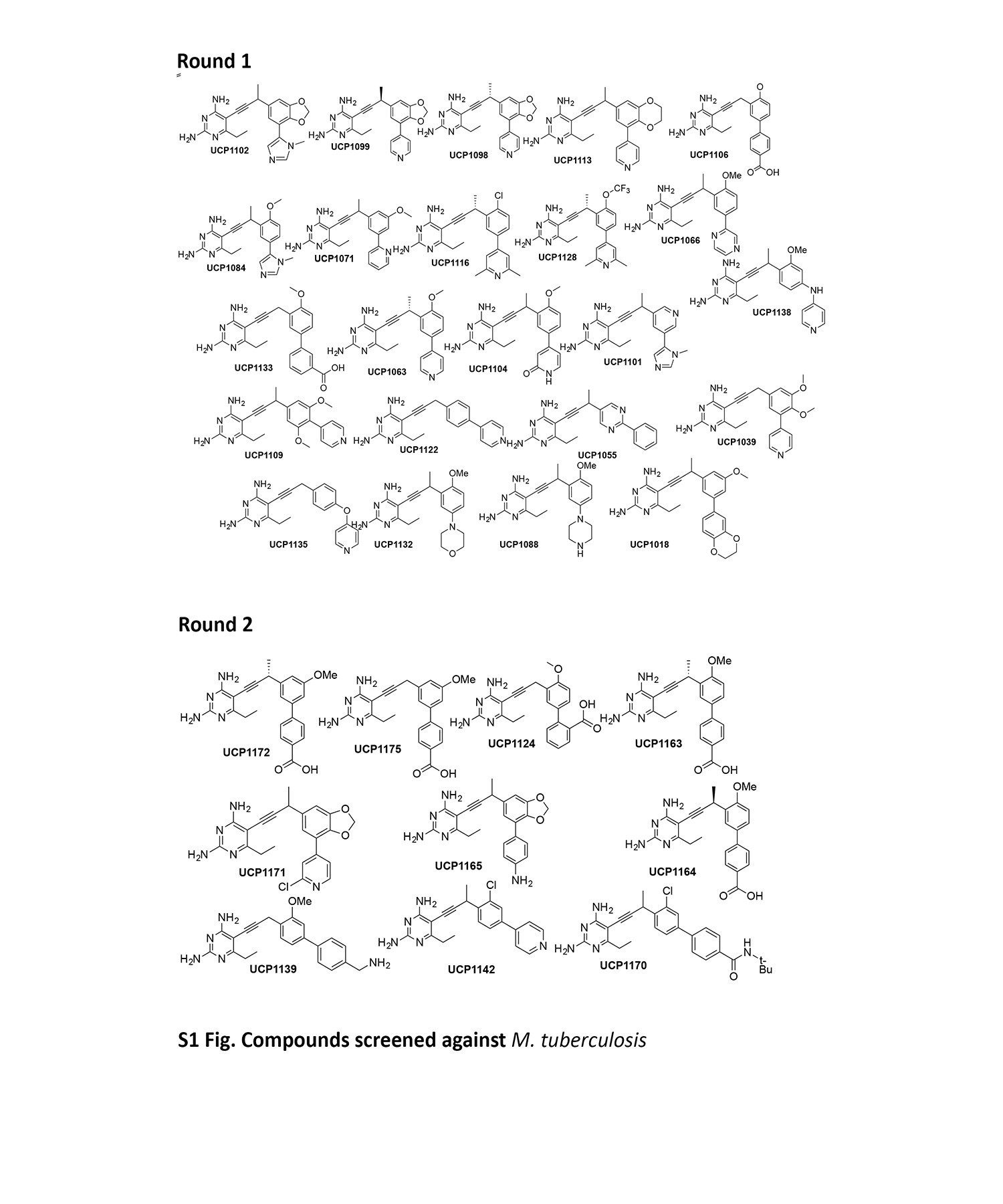

Supplement: S1 Fig — (TIF) [file pone.0161740.s001.tif]
