# Supplementary material for: Propargyl-Linked Antifolates Are Potent Inhibitors of Drug-Sensitive and Drug-Resistant Mycobacterium tuberculosis
Source: PLoS One. 2016 Aug 31;11(8):e0161740. doi: 10.1371/journal.pone.0161740 (PMC5006990; doi:10.1371/journal.pone.0161740)
Supplement: S1 Table — (DOCX) [file pone.0161740.s002.docx]

**S1 Table.** Propargyl-linked Antifolates Inhibit the Mtb DHFR Enzyme and Growth of Mtb

| **Compound** | **IC_50_ (nM)** | | **MIC (µg/mL)**  **Mtb** |
| --- | --- | --- | --- |
|  | **MtbDHFR** | **HuDHFR** |  |
| **Round 1** | | | |
| **UCP1102** | 326 ± 29 | 323 ± 27 | 2 |
| **UCP1106** | 173 ± 15 | 870 ± 33 | 4 |
| **UCP1098** | 206 ± 6 | 2914 ± 422 | 4 |
| **UCP1128** | 167 ± 1 | 168 ± 8 | 16 |
| **UCP1071** | 302 ± 33 | 2204 ± 226 | 4 |
| **UCP1113** | 1797 ± 179 | 290 ± 13 | 4 |
| **UCP1066** | 263 ± 4 | 233 ± 8 | 4 |
| **UCP1138** | 220 ± 37 | 290 ± 13 | 16 |
| **UCP1084** | 126 ± 6 | 1452 ± 193 | 2 |
| **UCP1116** | 114 ± 8 | 207 ± 9 | 8 |
| **UCP1099** | 122 ± 13 | 191 ± 14 | 8 |
| **UCP1133** | 311 ± 24 | 1577 ± 61 | 1 |
| **UCP1063** | 73 ± 6 | 144 ± 18 | 2 |
| **UCP1104** | 525 ± 64 | 625 ± 42 | 4 |
| **UCP1132** | 239 ± 4 | 229 ± 15 | 8 |
| **UCP1122** | 657 ± 92 | 1133 ± 76 | 8 |
| **UCP1109** | 333 ± 5 | 215 ± 18 | 8 |
| **UCP1088** | 302 ± 52 | 395 ± 21 | 16 |
| **UCP1055** | 249 ± 3 | 180 ± 15 | 32 |
| **UCP1101** | 582 ± 68 | 704 ± 23 | 16 |
| **UCP1135** | 249 ± 8 | 281 ± 34 | 16 |
| **UCP1039** | 297 ± 38 | 495 ± 58 | 32 |
| **UCP1018** | nd | 260 ± 20 | 16 |
| **Round 2** | | | |
| **UCP1172** | 177 ± 25 | 1015 ± 60 | <0.03 |
| **UCP1175** | 460 ± 50 | 688 ± 40 | 0.125 |
| **UCP1171** | 482 ± 20 | 254 ± 12 | 16 |
| **UCP1170** | 222 ± 21 | 175 ± 6 | 32 |
| **UCP1164** | 111 ± 7 | 1955 ± 46 | 0.5 |
| **UCP1163** | 102 ± 3 | 1632 ± 95 | 16 |
| **UCP1142** | 174 ± 9 | 106 ± 5 | 4 |
| **UCP1139** | 530 ± 40 | 284 ± 22 | 16 |
| **UCP1124** | 400 ± 44 | 10,081 ± 239 | 16 |
| **TMP** | 19,560 ± 200 | 97,179 ± 500 | 256 |
| **Trimetrexate** | 17^a^ | nd | 4 |
| **INH** | - | - | 0.03 |

^a^ Data from Nixon, et. al. {Nixon, 2014 #7}
