# Supplementary material for: Propargyl-Linked Antifolates Are Potent Inhibitors of Drug-Sensitive and Drug-Resistant Mycobacterium tuberculosis
Source: PLoS One. 2016 Aug 31;11(8):e0161740. doi: 10.1371/journal.pone.0161740 (PMC5006990; doi:10.1371/journal.pone.0161740)
Supplement: S2 Table — (DOCX) [file pone.0161740.s003.docx]

S2 Table. MIC values against *M. smegmatis* and over-expressing strains

| Compound | **MIC (µg/mL)** | | |
| --- | --- | --- | --- |
|  | *M. smegmatis*  (parent strain) | *M. smegmatis*  (Overexpressing DHFR Strain 1) | *M. smegmatis*  (Overexpressing DHFR Strain 2) |
| UCP1138 | 4 | 32 | 32 |
| UCP1128 | 0.5 | 128 | 64 |
| UCP1116 | 0.25 | 64 | 32 |
| UCP1113 | 1 | 64 | 32 |
| UCP1106 | 8 | >32 | >32 |
| UCP1102 | 1 | 64 | 32 |
| UCP1099 | 1 | 64 | 64 |
| UCP1098 | 0.5 | 64 | 32 |
| UCP1084 | 1 | 64 | 16 |
| UCP1071 | 4 | 128 | 64 |
| UCP1066 | 4 | 4 | 4 |
