# Supplementary material for: Propargyl-Linked Antifolates Are Potent Inhibitors of Drug-Sensitive and Drug-Resistant Mycobacterium tuberculosis
Source: PLoS One. 2016 Aug 31;11(8):e0161740. doi: 10.1371/journal.pone.0161740 (PMC5006990; doi:10.1371/journal.pone.0161740)
Supplement: S3 Table — (DOCX) [file pone.0161740.s004.docx]

S3 Table. Resistance pattern of organisms tested against DHFR inhibitors

MIC (µg/ml)

| Organism | INH | RIF | Ethambutol | Streptomycin | Moxifloxacin |
| --- | --- | --- | --- | --- | --- |
| Mtb Erdmann | 0.06 | 0.004 | 1 |  | 0.06 |
| Mtb 5 | 2 | 8 |  |  | 1 |
| Mtb 365 | 4 | 64 | 32 | 32 | 0.06 |
| Mtb 276 | 0.25 | 0.015 | 4 | 0.25 |  |
| Mtb 352 | 1 | 0.001 | 1, | 0.25 |  |
| Mtb 56 | 2 | 32 | 32 | 32 |  |
